# The human pathogenic 91del7 mutation in *SLC34A1* has no effect in mineral homeostasis in mice

**DOI:** 10.1038/s41598-022-10046-w

**Published:** 2022-04-12

**Authors:** Cornelia Bieri, Arezoo Daryadel, Carla Bettoni, Eva-Maria Pastor-Arroyo, Udo Schnitzbauer, Nati Hernando, Carsten A. Wagner

**Affiliations:** 1 Institute of Physiology, University of Zurich, Winterthurerstrasse 190, 8057 Zurich, Switzerland; 2 National Center of Competence in Research ‘NCCR Kidney.CH’, University of Zurich, Zurich, Switzerland

**Keywords:** Physiology, Kidney

## Abstract

Kidneys are key regulators of phosphate homeostasis. Biallelic mutations of the renal Na^+^/phosphate cotransporter *SLC34A1*/NaPi-IIa cause idiopathic infantile hypercalcemia, whereas monoallelic mutations were frequently noted in adults with kidney stones. Genome-wide-association studies identified *SLC34A1* as a risk locus for chronic kidney disease. Pathogenic mutations in *SLC34A1* are present in 4% of the general population. Here, we characterize a mouse model carrying the 91del7 in-frame deletion, a frequent mutation whose significance remains unclear. Under normal dietary conditions, 12 weeks old heterozygous and homozygous males have similar plasma and urinary levels of phosphate as their wild type (WT) littermates, and comparable concentrations of parathyroid hormone, fibroblast growth factor 23 (FGF-23) and 1,25(OH)_2_ vitamin D_3_. Renal phosphate transport, and expression of NaPi-IIa and NaPi-IIc cotransporters, was indistinguishable in the three genotypes. Challenging mice with low dietary phosphate did not result in differences between genotypes with regard to urinary and plasma phosphate. Urinary and plasma phosphate, plasma FGF-23 and expression of cotransporters were similar in all genotypes after weaning. Urinary phosphate and bone mineral density were also comparable in 300 days old WT and mutant mice. In conclusion, mice carrying the 91del7 truncation do not show signs of impaired phosphate homeostasis.

## Introduction

Phosphate is one of the most abundant anions and an essential mineral for all organisms. It is required for numerous cellular functions such as cell signalling, energy transfer, metabolic pathways, regulation of protein synthesis and activity, maintenance of acid–base homeostasis and bone mineralization. Plasma phosphate levels are determined by a balance between intestinal absorption of dietary phosphate, storage in the skeleton and urinary excretion through the kidneys^1–4^. The maintenance of phosphate homeostasis is achieved by interplay of an endocrine multi-tissue axis including kidney, parathyroid glands, intestine, and bone, responsible for transepithelial transport of phosphate and/or synthesis of phosphate regulatory hormones, namely parathyroid hormone (PTH), fibroblast growth factor-23 (FGF-23) and 1,25 (OH)_2_ vitamin D_3_^1^. 1,25 (OH)_2_ vitamin D_3_ stimulates the absorption of phosphate from the diet by upregulating the expression of NaPi-IIb/*SLC34A2*, without altering the paracellular pathway^5^. Low phosphate directly stimulates the production of 1,25 (OH)_2_ vitamin D_3_ by kidney cells in culture^3^ and in vivo^6^, through a mechanism that involves upregulation of Cyp27b1, the hydroxylase that converts 25-hydroxyvitamin D_3_ into the active 1,25 (OH)_2_ vitamin D_3_ metabolite, and downregulation of Cyp24a1, the enzyme that inactivates and degrades 1,25 (OH)_2_ vitamin D_3_. In contrast, high plasma phosphate levels increase the production and release of PTH^7,8^ and FGF-23^8^, which in turn reduce the expression of renal Na^+^/phosphate cotransporters NaPi-IIa (encoded by *SLC34A1*) and NaPi-IIc (encoded by *SLC34A3*) by promoting their internalization and degradation^9^. The hormonal reduction of renal phosphate cotransporters blunts renal phosphate reabsorption and contributes to normalize plasma phosphate levels^1^. Additionally, PTH stimulates renal synthesis of 1,25 (OH)_2_ vitamin D_3_ by a mechanism similar to the one described for low plasma phosphate^1,6^, whereas FGF-23 reduces 1,25 (OH)_2_ vitamin D_3_ levels by blunting its production and increasing its degradation^10,11^. FGF-23 performs its cellular specificity in the kidney and parathyroid glands by binding to its obligatory co-receptor α-klotho, a transmembrane protein that increases the affinity of FGF-23 to ubiquitously expressed FGF receptors^12^. FGF-23 suppresses PTH gene expression and secretion^13^, suggesting a regulatory feedback loop between FGF-23 and PTH. Additionally, 1,25 (OH)_2_ vitamin D_3_ enhances FGF-23 synthesis and blocks PTH production^14–16^. Dysfunction of these hormonal regulatory feedback loops or mutations of NaPi-IIa and NaPi-IIc, the two main phosphate transporters expressed in renal brush border membranes (BBM), have been shown to disturb phosphate balance and cause clinical disease phenotypes ^17–19^.

Mutations in *SLC34A3* are responsible for hereditary hypophosphatemic rickets with hypercalciuria (HHRH), a disorder characterized by reduced renal phosphate reabsorption and elevated 1,25 (OH)_2_ vitamin D_3_ level. HHRH patients display an increased risk of kidney stones and nephrocalcinosis^20,21^. Biallelic loss-of-function mutations in *SLC34A1* cause idiopathic infantile hypercalcemia (IIH) resulting from renal phosphate wasting, decreased circulating FGF-23 levels and increased 1,25 (OH)_2_ vitamin D_3_ production, causing a phenotype of hypercalcemia, hypercalciuria and nephrocalcinosis^17,22^. The 91del7 in-frame deletion at the N-terminus of NaPi-IIa is one variant among about 35 potential pathogenic mutations in the transporter that have been identified in IIH patients either as compound heterozygous or in homozygosity^22^. Moreover, monoallelic carriers were originally reported among patients with nephrolithiasis and renal phosphate loss^22^. However, this mutation is rather common and in large databases has been found in heterozygosity in about 2% of the general population^17,19^. The mutation arises from a 7 amino acid (21 bp) in-frame deletion that truncates amino acids 91–97 located within the N-terminal intracellular tail of the protein. Its functional implication remains unclear. Whereas some studies have indicated no link between the 91del7 mutation and phosphate handling/phosphate transport activities^22,23^, a report showed limited cell surface expression and transport activity of 91del7 mutation upon expression in *Xenopus laevis* oocytes and HKC-8 cells^19^. Moreover, at least one patient with biallelic 91del7 mutation developed full IIH disease symptoms^22^. In order to gain more insights into the in vivo relevance of this frequent variant, we have generated and characterized a mouse model carrying the 91del7 truncation in heterozygosity and homozygosity. Our data indicate no alterations in renal phosphate handling or systemic mineral metabolism.

## Results

### The 91del7 mutation results in viable mice and does not affect renal phosphate handling in adult mice fed standard diet

**Figure 1:**
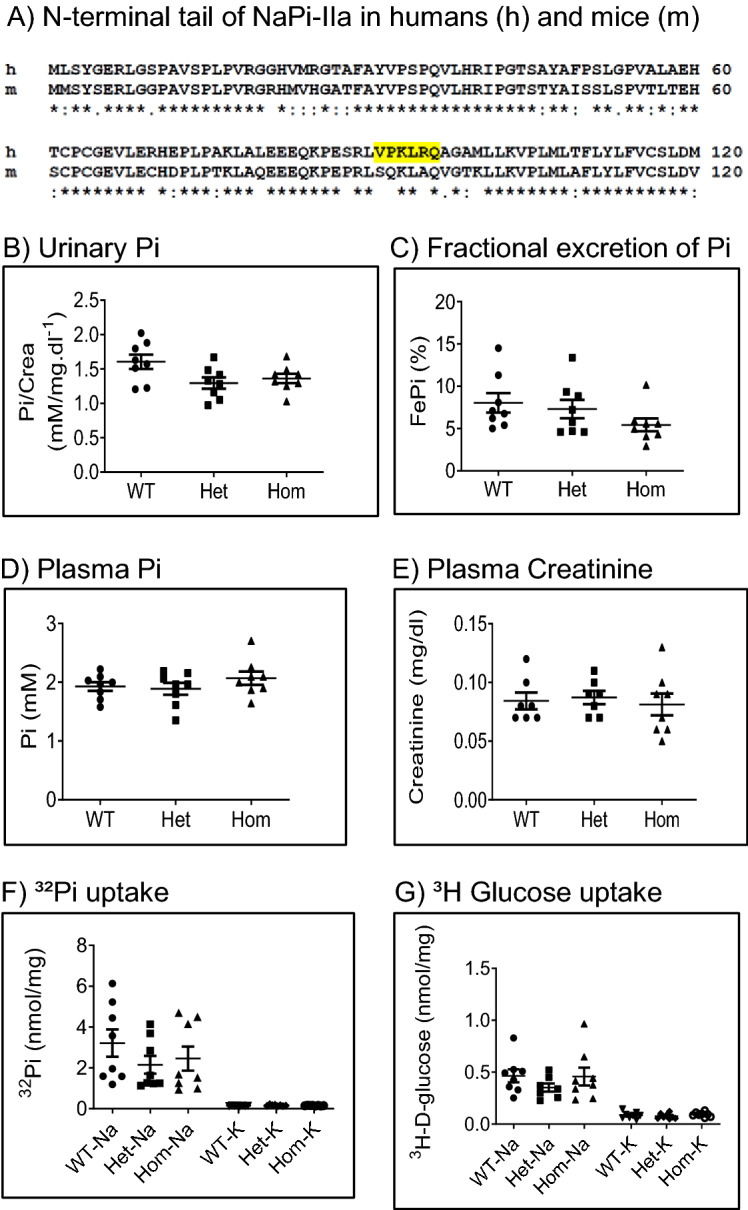
Generation of *Slc34a1*-91del7 mutant mice and effect on mineral balance. (**A**) Protein sequence comparison of the N-terminal tail of NaPi-IIa in humans and mice: numbers indicate amino acid position, the yellow shaded sequence is deleted in human patients with the *SLC34A1* 91del7 mutation and asterisks indicate fully conserved amino acids between humans and mice. (**B**, **C**) Urinary phosphate/creatinine ratio and fractional excretion of phosphate (FePi), (**D**, **E**) plasma levels of phosphate and creatinine. (**F**) ^32^Pi uptake and (**G**) ^3^H-D-glucose uptake obtained in kidney brush border membrane vesicles (BBMVs). Data are presented as Na^+^-dependent (first three columns) and Na^+^-independent (last three columns) values. All parameters were measured in samples from 12 weeks old wild type (WT), heterozygous (Het) and homozygous (Hom) 91del7 mice fed standard diet. Data are presented as single values together with mean ± SEM (n = 7–8 per group). Significance was tested by one-way ANOVA with Bonferroni correction.

CRISPR/Cas technology was used to delete in the mouse *Slc34a1* gene the nucleotide sequence corresponding to amino acids 91–97 of the NaPi-IIa protein (Fig. 1A). Care was taken to design gRNAs with low likelihood for off target binding. The deletion was verified by DNA sequencing and four founder mice were obtained. Because preliminary analysis identified 5 potential CRISPR/Cas off target sites, these sites were analyzed in founder mice by PCR amplification and sequencing. In one funder mouse, off target deletions were indeed identified and this mouse was not further used for establishing the mutant mouse line. Mutant heterozygous and homozygous mice were viable and fertile. Heterozygous mating produced litters with the expected Mendelian distribution of the three genotypes for both genders (data not shown). Urinary and plasma levels of phosphate, as well as plasma creatinine and fractional excretion of phosphate (FePi) were comparable in 12 weeks old male WT, Het and Hom mice fed standard diet (Fig. 1B–E), indicating that phosphate homeostasis is well preserved in 91del7 *Slc34a1* mice. Along the same line, analysis of Na^+^-dependent and Na^+^-independent phosphate transport activity into renal BBMV of WT, Het and Hom groups revealed no significant differences between the genotypes (Fig. 1F), suggesting that overall renal phosphate transport activity remains unchanged in 91del7 *Slc34a1* mice. No differences between groups were found either regarding the capacity of renal BBMVs to incorporate glucose (Fig. 1G). Additionally, coomassie blue staining of urinary samples and plasma urea concentration did not show any indication of kidney damage in Hom mice (Supplementary Fig. 1A,B).

### The 91del7 mutation does not change renal abundance of *Slc34* (NaPi-IIa and NaPi-IIc) and *Slc5a*1 transporters in adult mice fed standard diet

**Figure 2:**
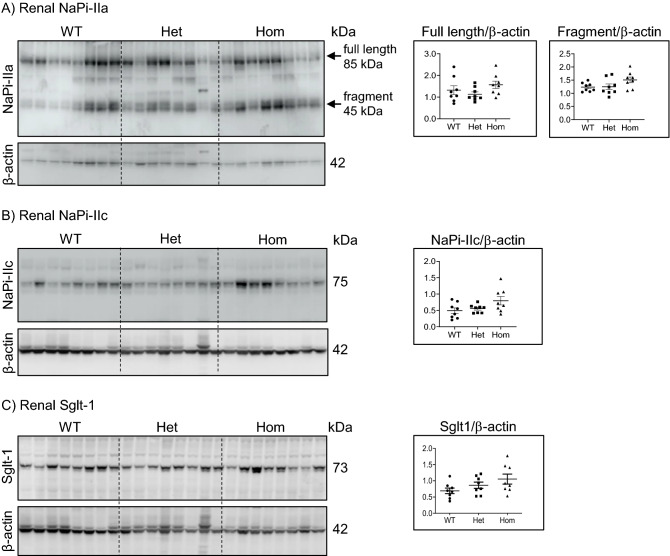
The 91del7 mutation has no effect on protein expression of phosphate (NaPi-IIa, NaPi-IIc) and glucose (Sglt-1) transporters in renal brush border membranes. (**A**) Protein abundance of the full-length and proteolytic fragment of NaPi-IIa, (**B**) protein abundance of NaPi-IIc and (**C**) protein abundance of Sglt-1 in kidney brush border membranes (BBM) of 12 weeks old wild type (WT), heterozygous (Het) and homozygous (Hom) 91del7 mice fed standard diet. The expression of the transporters was normalized to the corresponding β-actin. Data are presented as single values together with mean ± SEM (n = 8 per group). Significance was tested by one-way ANOVA with Bonferroni correction.

The protein abundance of the two major renal *Slc34* Na^+^/Pi cotransporters, NaPi-IIa and NaPi-IIc, was measured in the same renal BBMV preparations that were used for ^32^Pi uptake experiments. As shown in Fig. 2A, the expression of full-length NaPi-IIa (approx. 85 kDa) and its proteolytic fragment (approx.45 kDa) was unchanged between the groups. Similarly, NaPi-IIc abundance was comparable in all three genotypes (Fig. 2B). Additionally, protein expression of the Na^+^/glucose transporter Sglt-1 was comparable between the genotypes (Fig. 2C).

### The 91del7 mutation does not alter the PTH-FGF23 axis in adult mice fed standard diet

**Figure 3:**
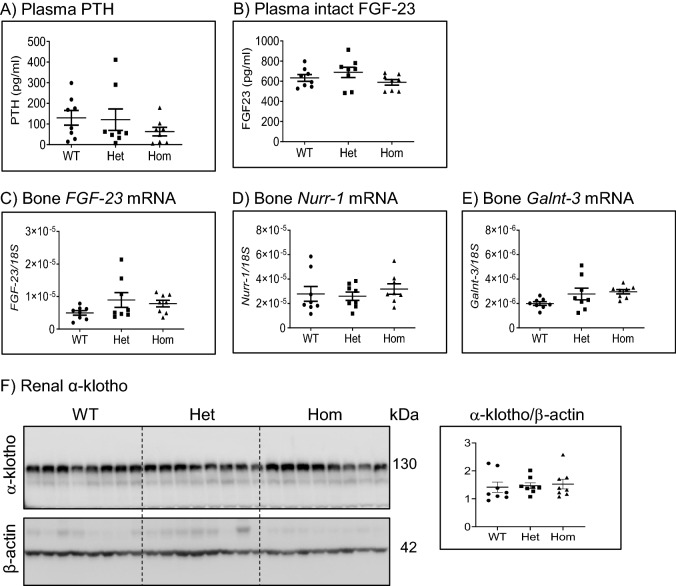
The 91del7 mutation has no effect on plasma FGF-23 and PTH levels, bone FGF-23 regulatory genes expression and renal protein expression of α-klotho. (**A**, **B**) Plasma levels of PTH and intact FGF-23. (**C**–**E**) Gene expression of *FGF23*, *Nurr-1* and *Galnt3* in femurs. Gene expression was normalized to the housekeeping gene *18S rRNA*. (**F**) Protein expression of α-klotho in renal homogenates. The protein abundance of α-klotho was normalized to β-actin. All parameters were analyzed in samples from 12 weeks old wild type (WT), heterozygous (Het) and homozygous (Hom) 91del7 mice fed standard diet. Data are presented as single values together with mean ± SEM (n = 8 per group). Significance was tested by one-way ANOVA with Bonferroni correction, *p* ≤ 0.05.

As shown in Fig. 3A, Hom and Het 91del7 *Slc34a1* mice had similar PTH values to WT when fed standard diet. Intact FGF-23 levels were also similar in all genotypes (Fig. 3B). Since systemic intact FGF-23 levels were comparable in all groups, the relative abundance of *Fgf-23* mRNA produced in bone was also comparable in all genotypes (Fig. 3C). Along the same line, mRNA expression of *Nurr-1*, the orphan nuclear receptor that activates *Fgf-23* gene expression upon PTH stimulation^24^, and *Galnt3*, the O-glycosylation mediator that modifies and prevents the proteolytic cleavage of FGF-23^25,26^ showed no differences between all three genotypes (Fig. 3D,E). In line with the comparable amounts of circulating FGF-23 levels and bone *Fgf23* mRNA expression, the renal protein abundance of α-klotho, the co-receptor of FGF-23, was similar in Hom, Het and WT groups (Fig. 3F).

### The 91del7 mutation does not affect calcitriol metabolism in adult mice fed standard diet

**Figure 4:**
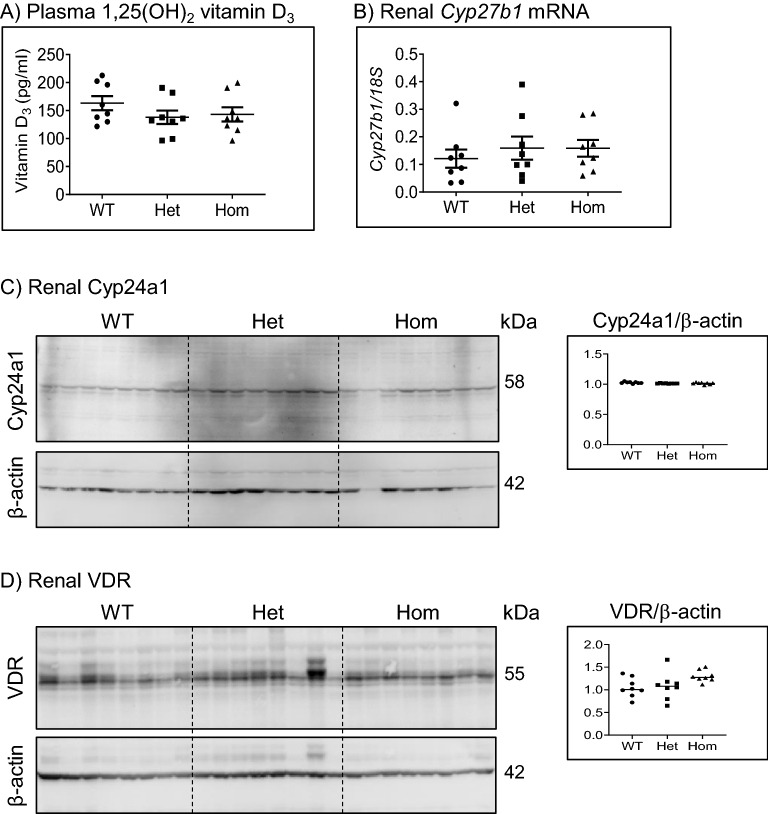
The 91del7 mutation has no effect on plasma 1,25-(OH)_2_ vitamin D_3_, renal *Cyp27b1* gene expression, and renal Cyp24a1 and VDR protein expression. (**A**, **B**) Plasma 1,25-(OH)_2_ vitamin D_3_ and renal gene expression of *Cyp27b1* normalized to the housekeeping gene *18S rRNA*. (**C**, **D**) Protein expressions of Cyp24a1 and VDR in kidney homogenates. The protein abundance of Cyp24a1 and VDR was normalized to the corresponding β-actin. All parameters were analyzed in samples from 12 weeks old wild type (WT), heterozygous (Het) and homozygous (Hom) 91del7 mice fed standard diet. Data are presented as single values together with mean ± SEM (n = 8 per group). Significance was tested by one-way ANOVA with Bonferroni correction.

Plasma levels of 1,25 (OH)_2_ vitamin D_3_ were similar in WT, Het and Hom mice groups fed standard diet (Fig. 4A). Additionally, the relative mRNA expression of the renal 1α-hydroxylase *Cyp27b1* was also comparable in all groups (Fig. 4B). Similarly, the renal protein abundance of the 24-hydroxylase Cyp24a1 was unchanged in WT and in 91del7 Het and Hom groups (Fig. 4C) in agreement with the similar systemic 1,25 (OH)_2_ vitamin D_3_ levels. In line with these data, renal VDR protein expression was not altered in the Hom group compared to Het and WT groups (Fig. 4D).

### The 91del7 mutation does not affect calcium homeostasis in adult mice fed standard diet

**Figure 5:**
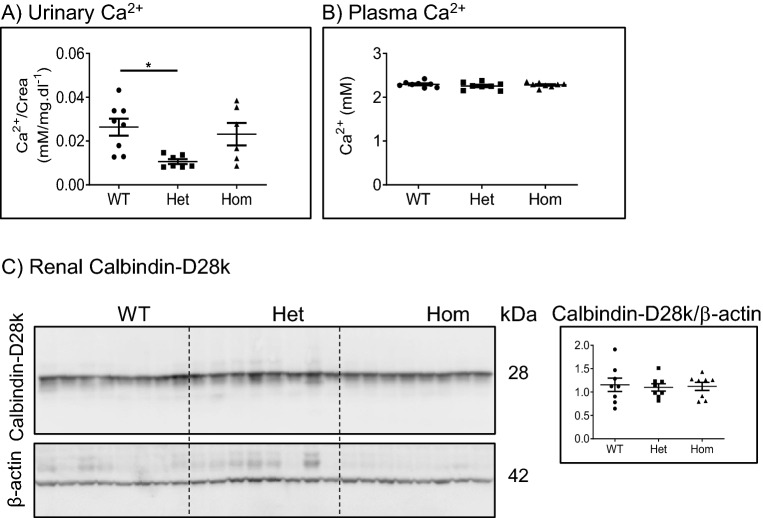
The 91del7 mutation has no effect on urinary and plasma calcium levels and renal calbindin-D28k protein expression. (**A**, **B**) Urinary and plasma levels of calcium. (**C**) Protein expression of calbindin-D28K in kidney homogenates. The protein abundance of calbindin-D28K was normalized to β-actin. All parameters were analyzed in samples obtained from 12 weeks old wild type (WT), heterozygous (Het) and homozygous (Hom) 91del7 mice fed standard diet. Data are presented as single values together with mean ± SEM (n = 7–8 per group). Significance was tested by one-way ANOVA with Bonferroni correction, *p ≤ 0.05.

The urinary and plasma levels of calcium were measured in samples from WT and 91del7 *Slc34a1* Het and Hom mice kept on standard diet (Fig. 5A,B). Apart from a mild decrease of urinary calcium in the Het group, the plasma and urine levels of calcium were comparable in all groups. The renal expression of the calcium-binding protein calbindin-D28k was also comparable in all three genotypes fed with standard diet (Fig. 5C), suggesting sustained calcium homeostasis in 12 weeks old 91del7 *Slc34a1* mice under standard diet.

### The 91del7 mutation does not alter phosphate homeostasis in young mice

**Figure 6:**
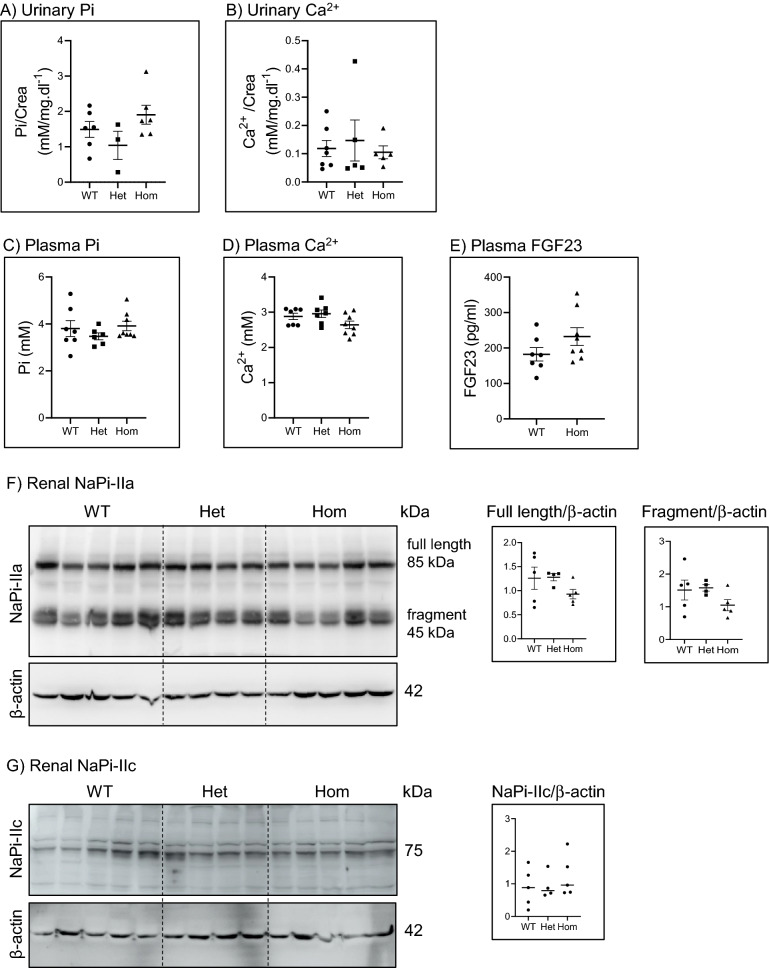
The 91del7 mutation has no effect on urinary and plasma parameters in young mice. (**A**, **B**) Phosphate and calcium concentration in spot urine obtained from 21 days old wild type (WT), heterozygous (Het) or homozygous (Hom) 91del7 mice. (**C**–**E**) Plasma phosphate, calcium and FGF-23 concentration in samples obtained from the same mice. (**F**, **G**) Expression of NaPi-IIa and NaPi-IIc in renal BBM from 21 days old pups from the three genotypes. The expression of the transporters was normalized to the corresponding β-actin. Data are presented as single values together with mean ± SEM (n = 3–7 per group). Significance was tested by one-way ANOVA with Bonferroni correction.

In humans, the alterations caused by mutations in *SLC34A1* are detected already in infants with idiopathic infantile hypercalcemia (IIH). Therefore, we also analyzed urinary and plasma metabolites in pups right after weaning. As shown in Figs. 6A–D, the levels of phosphate and calcium in spot urine and plasma were similar in 21 days old male pups of the three genotypes. Moreover, no differences in FGF-23 were found between WT and Hom pups right after weaning (Fig. 6E). The abundance of NaPi-IIa, both full length and proteolytic fragments, and NaPi-IIc was also comparable in BBM isolated from kidneys from WT, Het and Hom pups (Fig. 6F,G), all suggesting that the 91del7 truncation does not affect Pi homeostasis in young mice.

### The 91del7 mutation does not affect renal mineral excretion and bone in 300 days old mice fed standard diet

**Figure 7:**
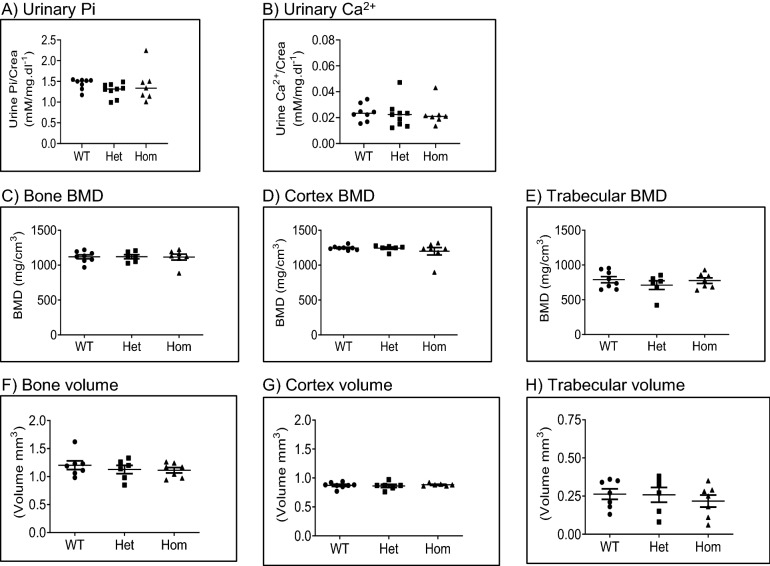
The 91del7 mutation has no effect on urinary excretion of phosphate and calcium and structural bone parameters in 300 days old mutant mice. (**A**,** B**) Urinary phosphate and calcium excretions normalized to creatinine. (**C**–**E**) Bone mineral density (BMD) of total bone, cortex or trabeculae. (**F**–**H**) Volume of total bone, cortex or trabeculae. All parameters were analyzed in samples obtained from 300 days old wild type (WT), heterozygous (Het) and homozygous (Hom) 91del7 mice fed standard diet. Data are represented as single values together with mean ± SEM (n = 6–8 per group). Significance was tested by one-way ANOVA with Bonferroni correction.

To test, whether older 91del7 *Slc34a1* mice have signs of age dependent disturbances of phosphate and calcium homeostasis, urinary phosphate and calcium excretion was assessed in 300 days old mice fed standard diet. As shown in Fig. 7A,B, both urinary excretion of phosphate and calcium were comparable between genotypes, further suggesting sustained phosphate and calcium homeostasis in 91del7 *Slc34a1* mice even at older age under standard diet. Moreover, bone mineral density (BMD) of 300 days old mice showed no significant differences between groups (Fig. 7C–E). Similarly, we found no differences between genotypes regarding bone volume and cortex remodeling (Fig. 7F–H).

### 91del7 mutant mice can adapt renal mineral excretion during dietary phosphate restriction

**Figure 8:**
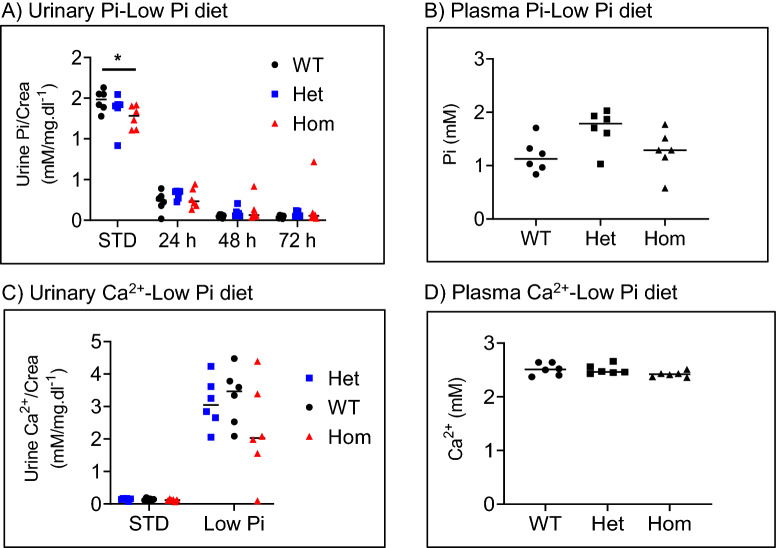
The 91del7 mutation has no effect on adaption to dietary phosphate restriction. (**A**) Urinary phosphate/creatinine ratio during one day of standard diet (STD) and upon switch to low phosphate diet for 1–3 days. (**B**) Plasma phosphate after 3 days of low phosphate diet. (**C**) Urinary calcium/creatinine ratio during one day of standard diet (STD) and upon 3 days of low phosphate (low Pi) diet. (**D**) Plasma calcium levels after 3 days of low phosphate diet. All parameters were analyzed in samples obtained from 12 weeks old wild type (WT), heterozygous (Het) and homozygous (Hom) 91del7 mice. Data are presented as single values together with mean ± SEM (n = 7–8 per group). Significance was tested by one-way ANOVA with Bonferroni correction, *p ≤ 0.05.

To determine whether mice carrying the 91del7 deletion can adapt to dietary phosphate restriction, 12 weeks old WT, Het and Hom mice were fed low phosphate diet for 4 days. Dietary phosphate restriction resulted in similar reductions of urinary phosphate excretion in WT and mutant mice (Fig. 8A). As shown in Fig. 8A,B, urinary and plasma phosphate levels were still similar in all groups upon dietary phosphate restriction. Feeding on low phosphate resulted in the expected increase in urinary excretion of calcium, an adaptation that was also comparable in the three genotypes (Fig. 8C). Like phosphate, urinary and plasma calcium levels were comparable in all genotypes upon restriction of dietary phosphate (Fig. 8C,D).

## Discussion

The importance of NaPi-IIa/*SLC34A1* and NaPi-IIc/*SLC34A3* for phosphate homeostasis is impressively underlined by the clinical problems seen in patients with inactivating mutations in these transporters leading to hypophosphatemia, hyperphosphaturia, hypercalcemia and –calciuria with kidney stones and nephrocalcinosis and in the case of NaPi-IIc also severe bone defects^1,17,18,22,27^. In humans, we and others identified homozygous and compound heterozygous mutations in *SLC34A1*, the gene encoding NaPi-IIa, and showed that these mutations affected trafficking of transporters into the membrane and transport function^22,27–29^. However, one mutation is of particular interest. A small in-frame deletion of seven amino acids at the intracellular N-terminus of NaPi-IIa (91del7) was identified in several patients as either one of two mutated alleles (compound heterozygous patients) or as homozygous mutation in at least one patient^22^. The symptoms of these patients were identical to patients with other *SLC34A1* pathogenic mutations, suggesting that the truncation impairs NaPi-IIa function in vivo. In contrast, in vitro analysis in Xenopus oocytes and OK cells demonstrated no obvious transport defect^22,23^ though expression of the mutant protein in OK and HKC-8 cells suggested reduced apical expression and partial retention, pointing to a possible trafficking defect^19,22^. The pathophysiologic relevance of the 91del7 variant in compound-heterozygous state is also supported by a report on an infant with Sotos syndrome and clinical IIH. The patient carried a genomic deletion of *SLC34A1* that is typical for Sotos syndrome on one allele and the 91del7 variant on the other *SLC34A1* allele^30^. However, this mutation in homozygous state revealed no obvious disturbance in phosphate metabolism and only borderline hypercalcemia and hypercalciuria, resembling a clinical characterization of mild IIH patients^22^.

Because it has been suggested that serum phosphate and possibly renal phosphate handling are genetically determined^31^ and interestingly, approximately 2% of the general population are heterozygous carriers of the 91del7 mutation^17,19^, we aimed to investigate the effect of this mutation in phosphate and calcium homeostasis in vivo. Our data shows no differences in plasma concentration and urinary excretions of phosphate and calcium between heterozygous and homozygous 91del7 mice and their WT littermates fed standard diet, not even immediately after weaning. However, targeted disruption of the entire NaPi-IIa gene in homozygous mice leads to increased urinary phosphate and calcium excretions, hypophosphatemia and hypercalcemia^22,32^. Likewise, a naturally occurring mutation in the mouse *Slc34a1* gene causes hyperphosphaturia, hypophosphatemia and hypercalcemia with hypercalcuria^33^. However, our results suggest a negligible impact of amino acids 91–97 of NaPi-IIa to renal handling of phosphate and calcium, at least in mice. In agreement with the similar plasma and urinary phosphate levels, phosphate transport capacity of renal BBMV, which is mediated mainly via NaPi-IIa and NaPi-IIc^34,35^, was comparable in 91del7 heterozygous and homozygous adult mice with WT, further suggesting that phosphate homeostasis is largely preserved in mice carrying the 91del7 mutation. These data are in line with previous work showing that phosphate uptake into *Xenopus* oocytes injected with cRNA encoding the human 91del7 truncated cotransporter is comparable to the phosphate incorporation into oocytes expressing the human WT protein^22^. Moreover, we have detected similar amounts of both NaPi-IIa (full and proteolytic fragments) and NaPi-IIc transporters in renal BBM of adult and young mice carrying 91del7 in heterozygosity and homozygosity and their WT siblings. In this regard, truncation of the whole N-terminal cytoplasmic tail of NaPi-IIa was found to have little effect on the sorting of the transporter when expressed in OK cells^36^. Consistently with the absence of changes in the expression of NaPi-IIa and NaPi-IIc in renal BBM, we found that systemic levels of PTH as well as intact FGF-23 remained unchanged in 91del7 mice compared with WT. PTH and FGF-23 are two major regulators of renal phosphate handling. Their levels increase in response to hyperphosphatemia/phosphate loading and are reduced under conditions that require phosphate retention^1^, pointing to their essential role on phosphate homeostasis. Considering that homozygous mice deficient for NaPi-IIa exhibit decreased PTH^32^, and FGF-23 levels^37^,the finding that circulating concentration of both hormones is normal in 91del7 mice, further suggest no role of residues 91–97 of the N-terminal intracellular tail of NaPi-IIa in renal phosphate handling and phosphate homeostasis. Constitutive ablation of *Slc34a1* causes hypercalcemia and hypercalciuria due to higher concentration of 1,25 (OH)_2_ vitamin D_3_ in plasma^22,32,35,37^. Renal production of 1,25 (OH)_2_ vitamin D_3_ controls and it is controlled by PTH, FGF-23, phosphate and calcium^1^. The higher 1,25 (OH)_2_ vitamin D_3_ reported in constitutive *Slc34a1*-deficient mice is likely due to a combination of reduced catabolism and increased synthesis as the mRNA levels of *Cyp24a1* are reduced, whereas those of *Cyp27b1* are increased in mutant mice^22^. In addition to the mouse model, IIH patients with NaPi-IIa mutations exhibit a primary defect in proximal tubular phosphate reabsorption. Subsequent hypophosphatemia induces a decrease in systemic FGF-23 levels^38,39^. Both hypophosphatemia and low FGF-23 levels increase Cyp27b1 and inhibit Cyp24a1 expressions and activities^38^. These effects together promote an increase of 1,25 (OH)_2_ vitamin D_3_ with subsequent hypercalcemia. As a consequence of no change in PTH, FGF-23, phosphate and calcium levels in 91del7 mice, plasma concentration of 1,25 (OH)_2_ vitamin D_3_ and renal expressions of *Cyp27b1*, Cyp24a1 and 1,25 (OH)_2_ vitamin D_3_ receptor VDR were comparable in 91del7 adult mutant mice and their WT controls. In addition to the urinary and plasma alterations described above, young *Slc34a1* homozygous mutants mice exhibited poorly developed trabecular bones and retarded secondary ossification which improved with age^32^. However, another study did not find any bone parameters changes in 12 weeks old NaPi-IIa knockout mice^40^. Along the same line, 300 days old 91del7 mice exhibited similar urinary and plasma phosphate and calcium levels than WT and showed no change of bone mass and structural bone parameters.

Considering the preserved phosphate and calcium homeostasis observed in young and adult 91del7 mice fed standard diet, next we challenged them with low phosphate diet and monitored urinary phosphate and calcium levels at three constitutive days. Importantly, low phosphate diet led to similar reductions in urinary excretion of phosphate and increase in urinary output of calcium in 91del7 homozygous and heterozygous mice and their WT littermates, further evidencing the fully maintained phosphate and calcium homeostasis in 91del7 mice under different diet conditions. Also, plasma phosphate and calcium levels were similar between genotypes. Thus, 91del7 mice do not exhibit any overt systemic phosphate and calcium disturbances, which slightly differs from the observation reported in patients carrying the 91del7 mutation. Whether this discrepancy represents a species difference or whether additional factors in patients are required to uncover the phenotypic defect, remains to be established.

Taken together, our data indicate that phosphate and calcium homeostasis are not impaired in 91del7 variant mice at different dietary conditions, as none of the relevant parameters was changed. While both the N^41^ and C-terminal^42^ cytoplasmic tails of NaPi-IIa have been reported to interact with a number of intracellular and transmembrane proteins, only associations with the C-terminal tail are known to control its apical expression^43^. Furthermore, the N-terminal tail does not seem to be required for proper apical expression of the transporter in vitro. Therefore, further studies are required to reconcile this in vitro data and the present in vivo results with the suggested pathogenicity of the 91del7 truncation in humans.

## Materials and methods

### Mice

Mice mimicking the 91del7 mutation identified in human patients were generated by Cyagen (Santa Clara, California, USA) using CRISPR/Cas technology in the C57BL/6 background. 12 weeks old wild type (WT), heterozygous (Het) or homozygous (Hom) male mice were fed either constantly with standard diet (0.8% Pi, 1% Ca and 1000 IU/kg vitamin D_3_) (Kliba #3436) or with low phosphate diet (0.1% Pi, 1% Ca and 1000 IU/kg vitamin D_3_) (Kliba #2169) for 4 days, and received water ad libitum. Animals were placed into metabolic cages (Mouse diuresis cages, Tecniplast, Italy) for the last 3 days and water and food intake as well as urine production and body weight were measured. Urine was collected under mineral oil and stored at − 20 °C for ion measurements. After 3 days, animals were anaesthetized with isoflurane and venous blood was taken, centrifuged (8000 rpm, 10 min at 4 °C) and the plasma was stored at − 80 °C. Then, kidneys and femurs were collected and stored at − 80 °C. Plasma, spot urine and kidneys were also collected from 21 days old mice (immediately after weaning). In a third experiment, mice were fed on a normal diet until reaching 300 days, upon which plasma, 24 h urine (in metabolic cages) and femurs (for micro-computed tomography) analysis were collected.

All procedures were carried out according to the Swiss animal welfare laws and guidelines for animal care and were approved by the Veterinary Office of the Canton Zurich (license number 156/2016).

### Determination of urinary and plasma metabolites

Urinary and plasma levels of calcium, phosphate, creatinine and urea were measured with a UniCel DxC 800 Synchron Clinical System (Beckman Coulter) in the Zurich Integrative Rodent Physiology (ZIRP) facility. Fractional excretion of phosphate (FEPi) was calculated as ((urinary phosphate x plasma creatinine)/(plasma phosphate × urine creatinine)) × 100.

Plasma levels of intact FGF-23 (Immutopics; 60–6800), PTH (Immutopics; 60-2305) and 1,25 (OH)_2_ vitamin D_3_ (Immunodiagnostic Systems (IDS)) were determined by Elisa and radioimmunoassay techniques, respectively, according to the manufactures’ protocols. Urinary proteins were detected by coomassie blue of SDS-Page gel loaded with urine samples normalized to 5 µg creatinine; bovine serum albumin (BSA, 0.5 mg/ml) was loaded as a positive control.

### RNA isolation and semi-quantitative Real-Time PCR (qRT-PCR)

RNA was isolated from kidney and femur samples using the Qiagen RNeasy Mini extraction kit (Qiagen, Germany) according to instructions provided by the manufacturer. Purified RNA was transcribed to cDNA (TaqMan Reverse Transcription Kit, Applied Biosystems) which was later used as template for the PCR. The relative expression of the genes of interest was quantified by using sequence-specific forward and reverse primers together with sequence-specific probes labeled with reporter (5′-end, FAM) and quencher (3′-end, TAMRA) dyes. Custom-designed primers and probes were synthetized by Microsynth (Balgach, Switzerland) whereas commercially available primers were purchased from Applied Biosystems (Supplementary Table 1). Ribosomal 18S RNA (Applied Biosystems) was used as housekeeping gene. The relative fold change was calculated according to the formula 2^(Ct (18 s)−Ct (gene of interest))^.

### Measurements of ^32^Pi and ^3^H-Glucose fluxes into renal brush border membrane vesicles (BBMV)

Renal BBMVs were prepared according to the Mg^2+^ precipitation technique^44^. Briefly, one kidney was cut into small pieces and placed in cold homogenization buffer (300 mM D-mannitol, 5 mM EGTA and 12 mM Tris–HCl, pH 7.1). Tissue was homogenized for 2 min on ice using the polytron PT 1200 E (Kinematica, Lucerne, Switzerland). The homogenate was diluted with cold water and the solution mixed before an aliquot was taken and stored at − 20 °C for immunoblotting experiments. MgCl_2_ (final concentration 12 mM) was added to the rest of the homogenate for precipitation of non-apical membranes. Samples were left on ice for 15 min before being centrifuged at 4500 rpm for 15 min at 4 °C. The supernatant was collected and centrifuged at 18,000 rpm for 30 min at 4 °C. The pellet containing the BBMVs was then resuspended in a membrane buffer, consisting of 300 mM D-mannitol and 20 mM HEPES-Tris pH 7.4, with the help of a syringe and a 25G needle. The suspension was centrifuged once more at 18,500 rpm for 30 min at 4 °C before the pellet was resuspended again in the membrane buffer to obtain a homogenous suspension. The BBMVs were directly used for flux measurements and the remainders kept at − 20 °C for immunoblotting experiments.

Uptake of ^32^P-labelled phosphate and ^3^H-D-glucose were measured according to the filtration technique^44^, by incubating BBMV in two different solutions, containing 100 mM Mannitol and 20 mM HEPES-Tris, pH 7.4 and either 125 mM NaCl or 125 mM KCl. These two solutions were each supplemented with either 0.125 mM H_2_PO_4_ pH 7.4 containing ^32^Pi as a tracer, or with 0.125 mM D-glucose and ^3^H-D-glucose as a tracer. Uptakes were performed at 25 °C for 30 s or 2 h. After the incubation time, uptakes were stoped by addition of cold stop solution (100 mM Mannitol, 5 mM Tris–HCl pH 7.4, 150 mM NaCl, 5 mM Pi and 5 mM glucose). The suspension was spotted onto cellulose filters (0.45 μm), and the filters vacum-washed and transferred to scintillation vials. Upon addition of 4 ml of scintillation media (Emulsifier-Safe, 6013389, PerkinElmer, Groningen, The Netherlands); the incorporation of radioactivity was measured with a β-counter (Tri-Carb 2900TR liquid scintillation analyzer, Packard BioScience, Connecticut, USA). All measurements were done in triplicates. To obtain the Na^+^ dependent uptakes, values measured with the K^+^ medium were substracted form the values obtained with the Na^+^ medium.

### Western blot

Western blots were performed with renal BBM (prepared for the fluxes experiments in the case of adult mice) as well as with total proteins obtained by homogenization in RIPA buffer containing 50 mM Tris–HCl (pH 7.4), 150 mM NaCl, 1% NP-40, 0.5% deoxycholate acid sodium salt supplemented with phenylmethylsulfonyl fluoride (PMSF) and protease inhibitor cocktail (Roche, Basel, Switzerland). Samples of either total or BBMV proteins were separated by electrophoresis in 8%-10% SDS–polyacrylamide gels and transferred to PVDF membranes (EMD Millipore, Billerica, MA). After blocking with 5% powdered milk in 0.1% TBS-tween buffer, membranes were incubated overnight at 4 °C with primary antibodies (Supplementary Table 2). After extensive washing with TBS-tween buffer, membranes were further incubated with appropriate secondary antibodies (Supplementary Table 2) for 1 h at room temperature and finally were exposed to chemiluminescent substrate. Protein signals were detected on a LAS-4000 Luminescent Image Analyzer and the signals were quantified with Advanced Image Data Analyzer (AIDA; Raytest). The expression of the proteins of interest was normalized to the abundance of β-actin.

### Micro-computed tomography

The distal epiphysis of femurs from 300 days old mice was scanned using the Quantum FX microCT Imaging System (PerkinElmer, Waltham, MA). The bones were placed in the micro CT and were scanned at a current of 100 µA and 90 kV voltage with a 5 mm field of view and a total scan time of 3 min for 360°. Scan analysis was done with the program Analyze 12.0 and the bone microarchitecture analysis (BMA) add-on module (AnalyzeDirect, Inc., Overland Park, Kansas, USA). Only 100 sections from the epiphyseal plate into the diaphysis were considered for the analysis. To convert the CT numbers (Hounsfield units) to bone mineral density (mg/CC units) a hydroxyapatite phantom of known density was scanned, and a calibration curve generated. For analysis, a slope (SigmaCT) value of 0.8011 and an offset (BetaCT) value of 1651.3 were used.

### Statistics

Data are represented as mean ± standard error of mean (SEM). Significance was tested using a one-way ANOVA with Bonferroni post-hoc correction. P values smaller than 0.05 were considered statistically significant. Softwares used for analysis were Fiji/ImageJ (Madison, Wisconsin, USA) and GraphPad prism 5 (GraphPad Software, San Diego, US). The study is reported in accordance with ARRIVE guidelines.

## Supplementary Information

Supplementary Legends.

Supplementary Information 2.
