# Supplementary material for: The human pathogenic 91del7 mutation in SLC34A1 has no effect in mineral homeostasis in mice
Source: Sci Rep. 2022 Apr 12;12:6102. doi: 10.1038/s41598-022-10046-w (PMC9005600; doi:10.1038/s41598-022-10046-w)
Supplement: Supplementary file 1 — Supplementary Legends. [file 41598_2022_10046_MOESM1_ESM.docx]

**Supplementary figure 1. The 91del7 mutation has no effect on renal function.**

(A) Coomassie blue staining of SDS-Page loaded with urine samples and BSA as control, and (B) plasma urea obtained from samples of 12 weeks old wild type (WT), heterozygous (Het) or homozygous (Hom) 91del7 mice fed standard diet. Data are presented as mean ± SEM (n=3-8 per group). Significance was tested by one-way ANOVA with Bonferroni correction, p≤0.05.

**Supplementary figures 2-9**

Original Western blots shown in Figures 2-6

**Supplementary table 1. Sequences of** Primers and probes used for qPCR.

**Supplementary table 2.** Primary and secondary antibodies used for western blot
