# Supplementary material for: The human pathogenic 91del7 mutation in SLC34A1 has no effect in mineral homeostasis in mice
Source: Sci Rep. 2022 Apr 12;12:6102. doi: 10.1038/s41598-022-10046-w (PMC9005600; doi:10.1038/s41598-022-10046-w)
Supplement: Supplementary file 2 — Supplementary Information 2. [file 41598_2022_10046_MOESM2_ESM.pdf]

Supplementary Figure 1

A) Urine coomasie blue

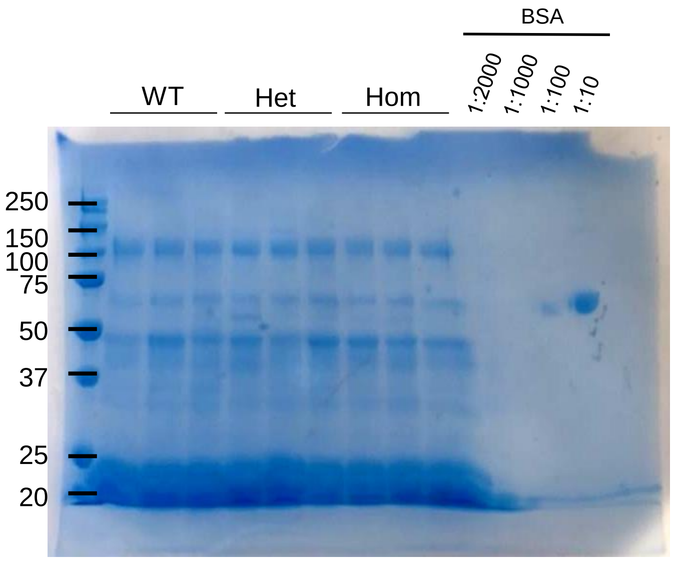

B) Plasma Urea

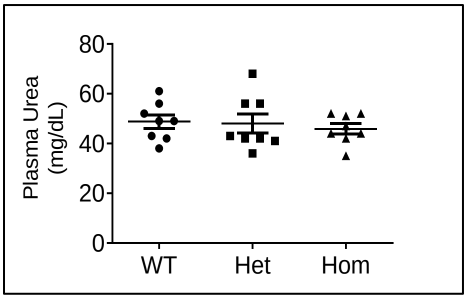

Supplementary Figure 2: full NaPi-IIa and β-actin Western blots shown in Figure 2A

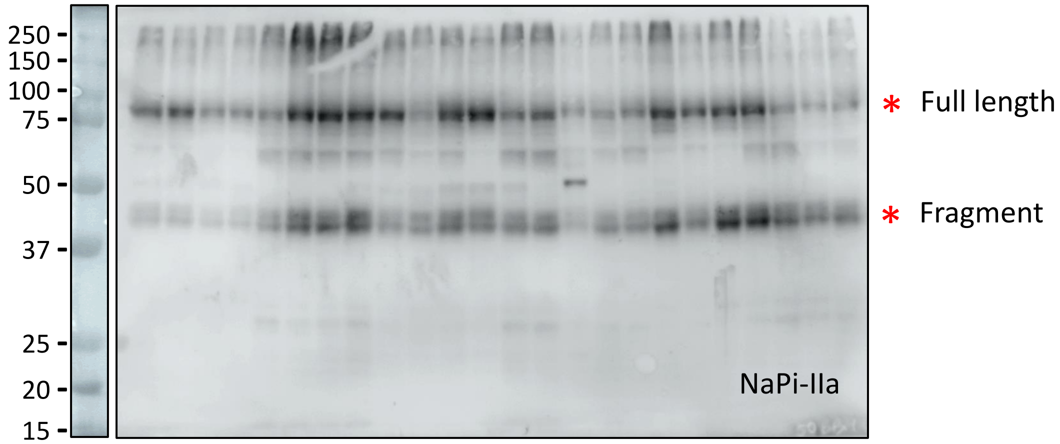

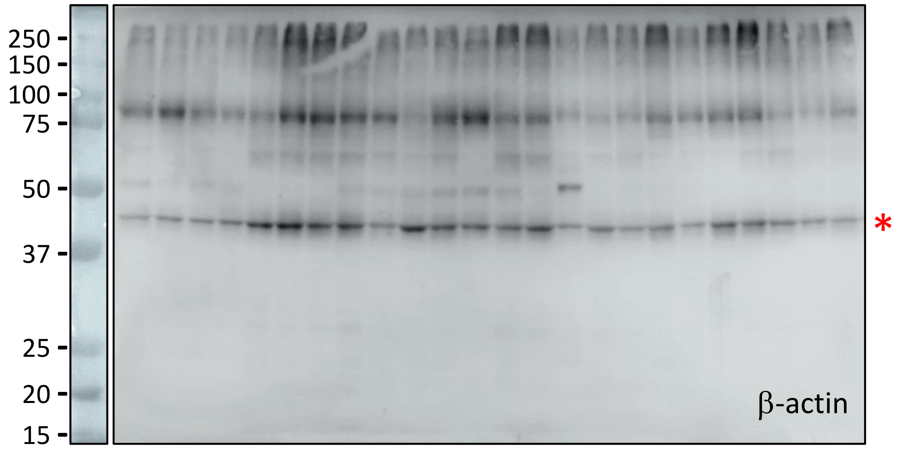

Supplementary Figure 3: full NaPi-IIc and β-actin Western blots shown in Figure 2B

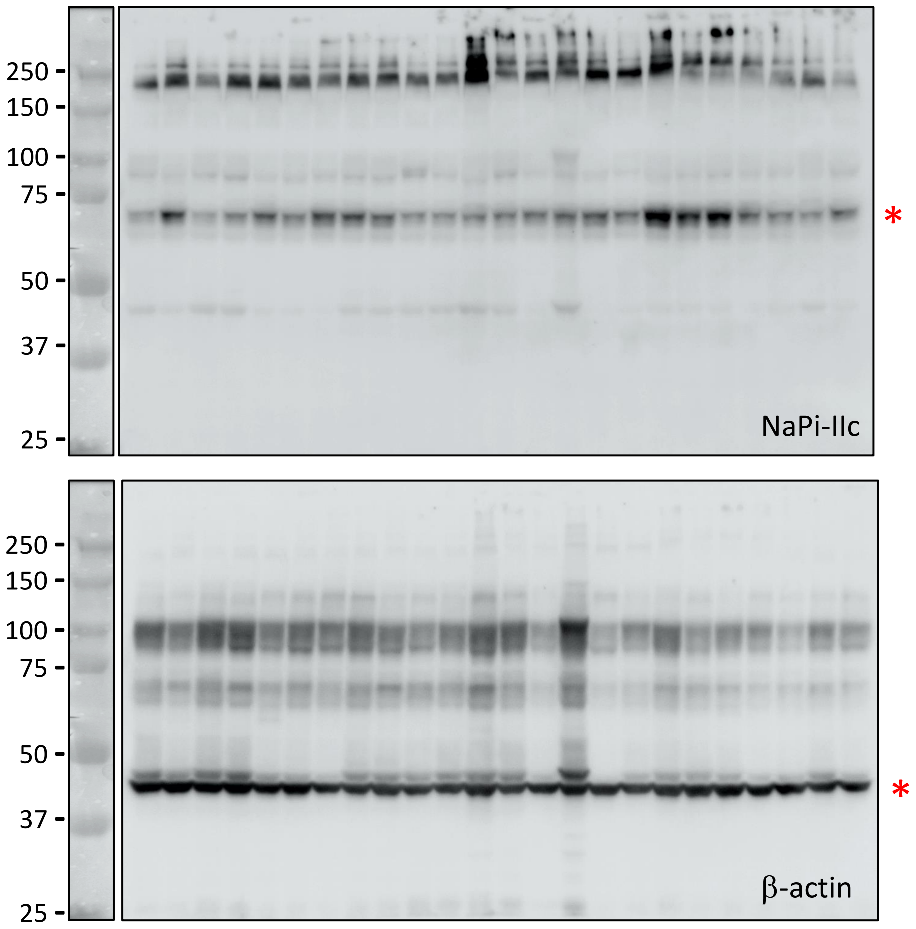

Supplementary Figure 4: full Sglt1 and β-actin Western blots shown in Figure 2C

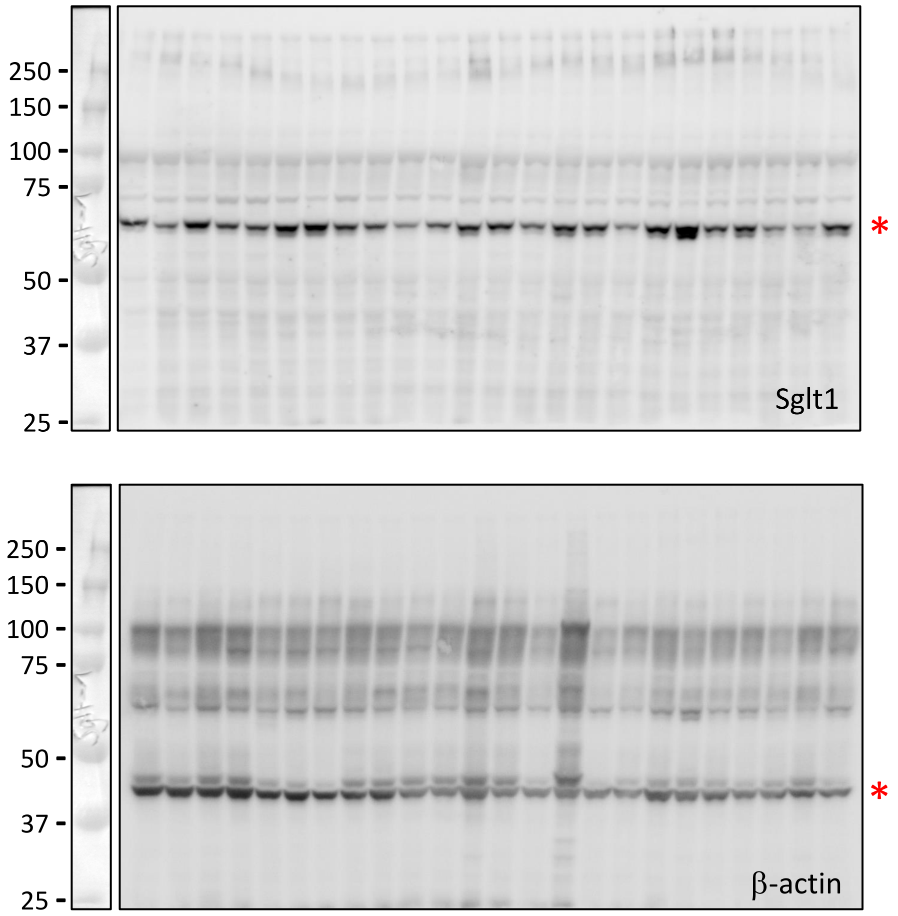

Supplementary Figure 5: full α-klotho and β-actin Western blots shown in Figure 3F

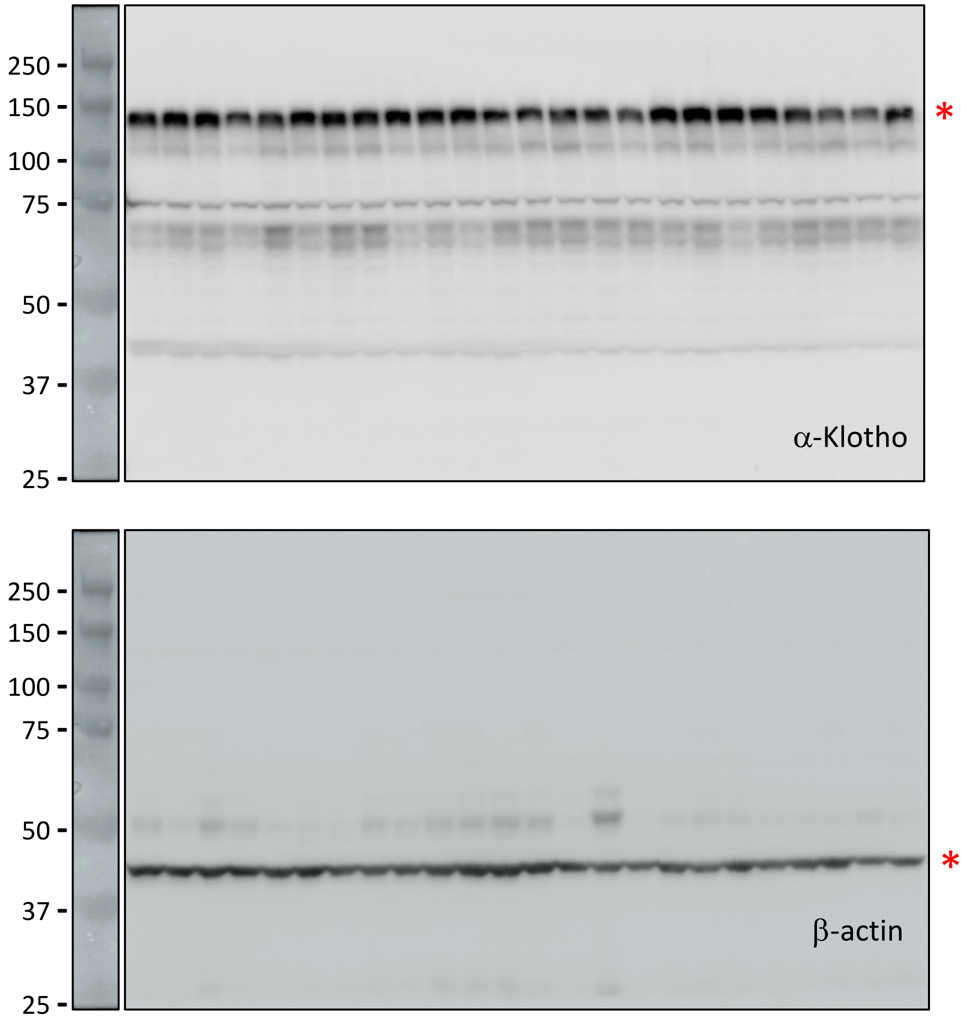

Supplementary Figure 6: full Cyp24a1 and β-actin Western blots shown in Figure 4C

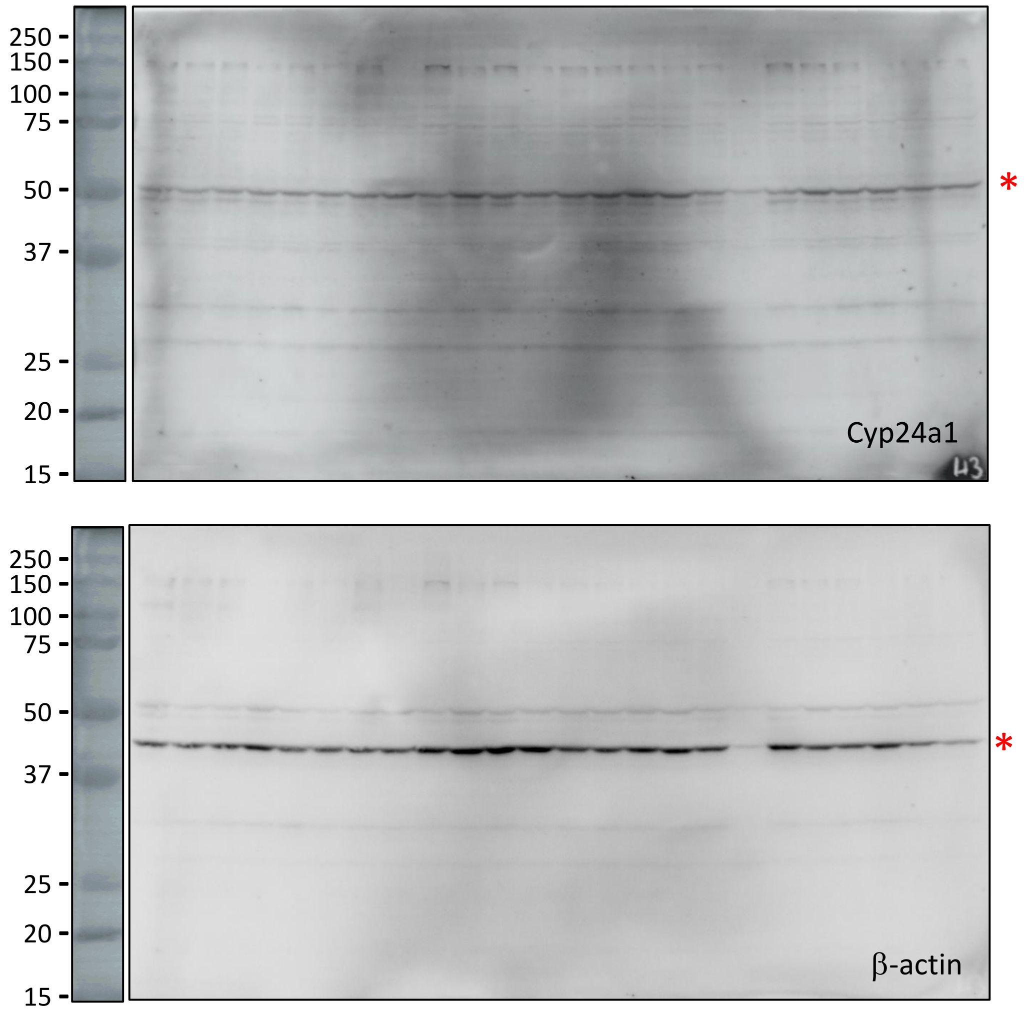

Supplementary Figure 7: full VDR and β-actin Western blots shown in Figure 4D

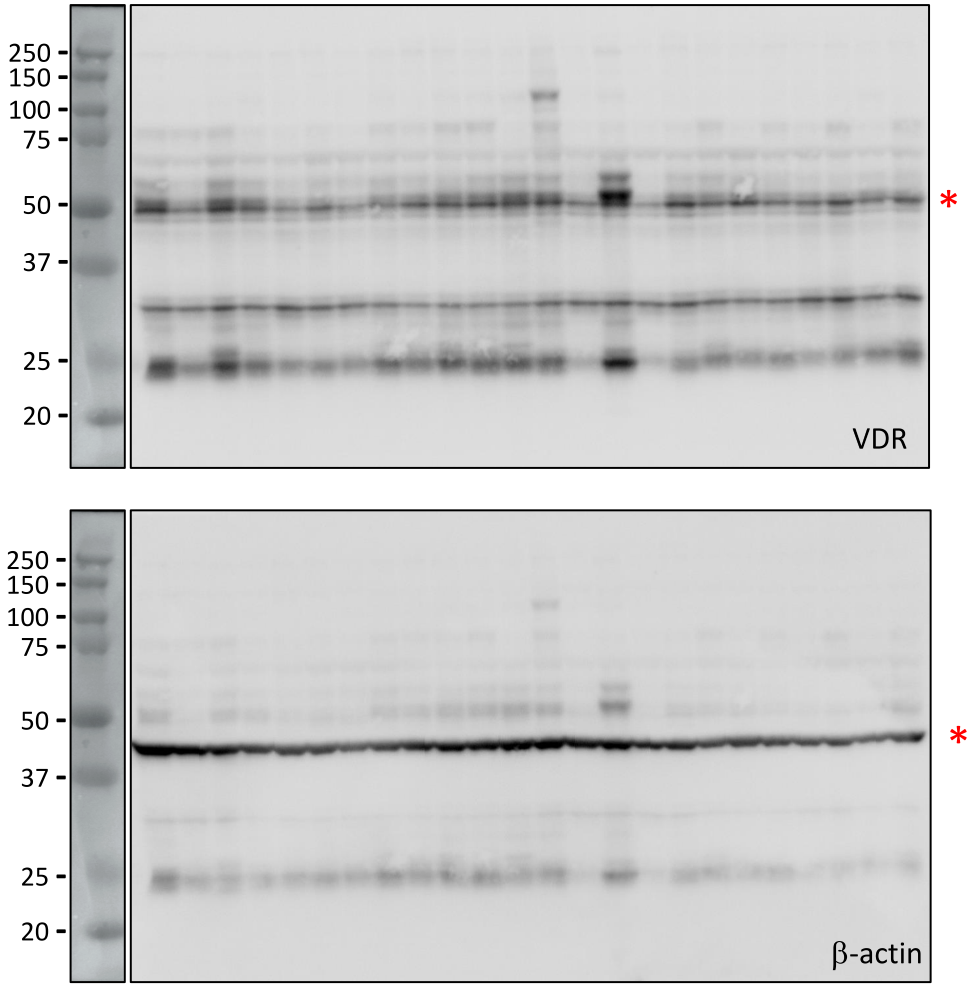

Supplementary Figure 8: full calbindin-D28k and β-actin Western blots shown in Figure 5C

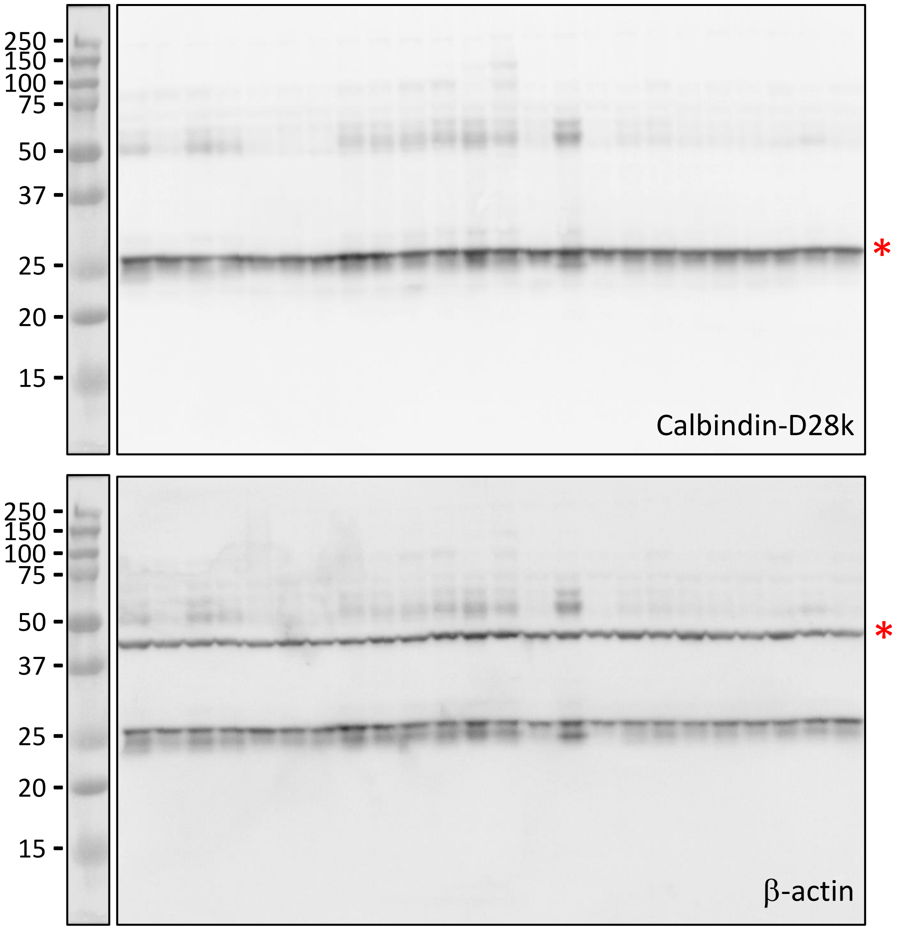

Supplementary Figure 9: full NaPi-IIa, NaPi-IIc and β-actin Western blots shown in Figure 6F

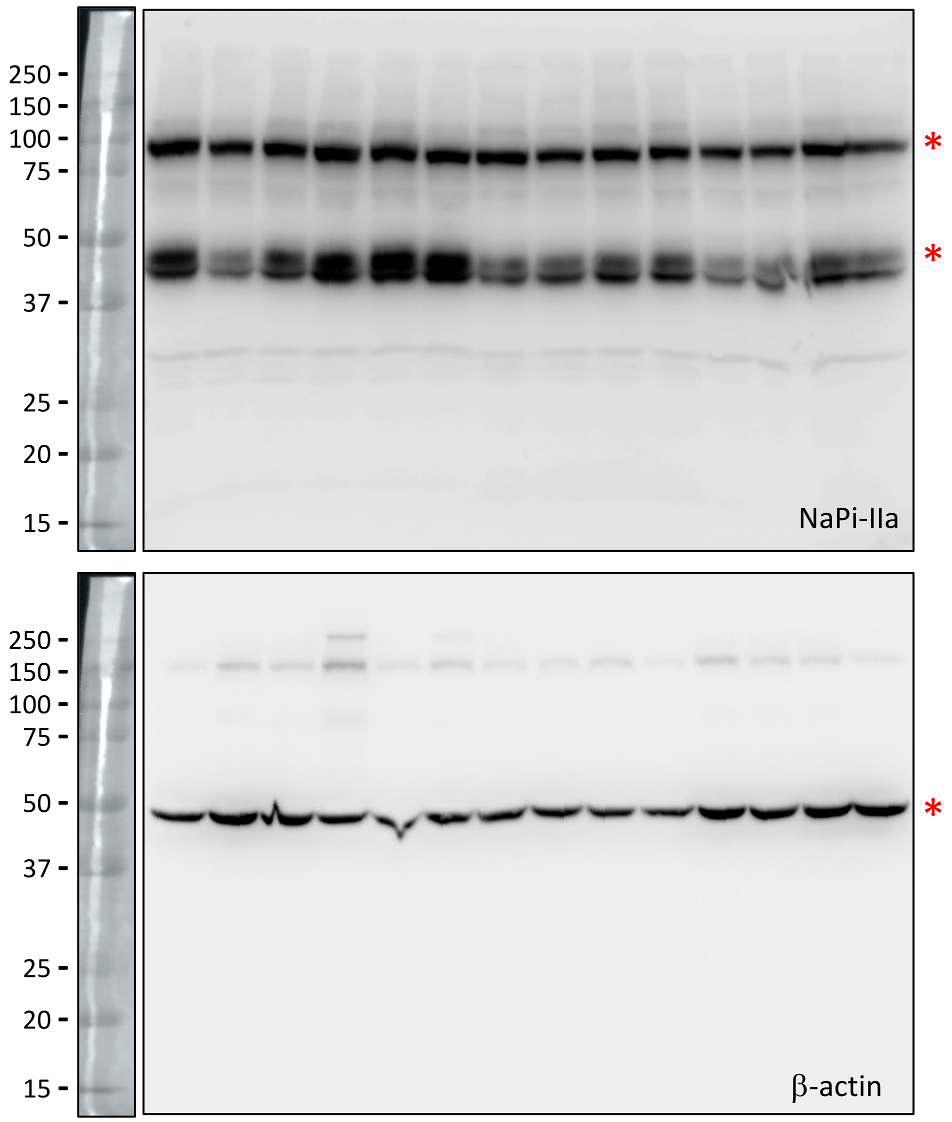

Supplementary Figure 9: full NaPi-IIc and β-actin Western blots shown in Figure 6G

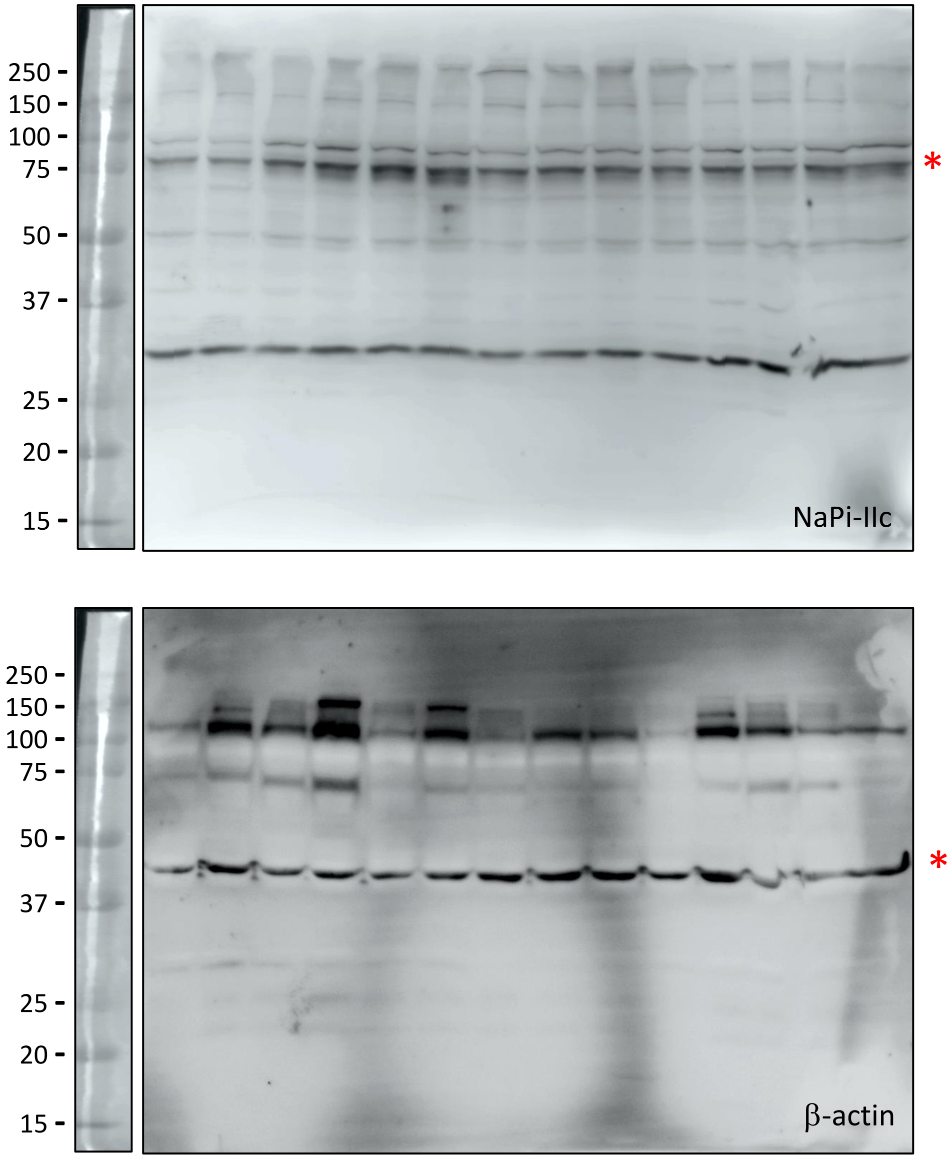

Supplementary table 1: sequences of primers and probes

Gene	Primer (5’-3’)	Probe (5’-3’)
**18s RNA**	Eukaryotic 18S rRNA Endogenous Control (4310893E, Applied Biosystems™, Thermo Fisher Scientific)	
**FGF23**	Forward: TCA GAC CAT CTA CAG TGC CCT<br>Reverse: GGA ACC TTC GAG TCA TGG CT	AGA GGA CGC CGG CTC TGT GGT GAT A
**Galnt3**	Forward: GAG AAA GAG CGA GGG GAA AC<br>Reverse: GTG GAC CAT GCT TCA TTG TG	ACA CCC GAC CAC CTG AAT GTA TTG AAC
**Nurr-1**	Forward: CAT CGA CAT TTC TGC CTT CTC<br>Reverse: CTT CCA CTC TCT TGG GTT CCT	TGC CCT GGC TAT GGT CAC AGA GAG A
**Cyp27b1**	Mm01165912_g1 (Applied Biosystems™, Thermo Fisher Scientific)	

Supplementary table 2: Primary and secondary antibodies used for western blot

Primary antibodies	Species	Dilution	Expected size (Kda)	Reference/Company
Anti-NaPi-IIa	Rabbit	1:2000	75-100	[(Biber, Stieger et al. 2007)]
Anti-NaPi-IIc	Rabbit	1:2000	75-100	[(Nowik, Picard et al. 2008)]
Anti-Sglt-1	Rabbit	1:1000	73	Abnova
Anti-Cyp24a1	Rabbit	1:1000	54-59	Protein Tech
Anti-VDR	Mouse	1:1000	48-60	Santa Cruz
Anti-calbindin-D28K	Mouse	1:1000	28	Swant
Anti-α-klotho	Rat	1:1000	130	TransGenic Inc.
Anti-β-actin	Mouse	1:5000	42	Sigma-Aldrich
**Secondary antibodies**		**Dilution**		**Company**
Anti-mouse (AP)		1:5000		Promega
Anti-rabbit (AP)		1:5000		Promega
Anti-rat (HRP)		1:5000		R&D systems
